# IL-4 Alleviates Ischaemia-Reperfusion Injury by Inducing Kupffer Cells M2 Polarization via STAT6-JMJD3 Pathway after Rat Liver Transplantation

**DOI:** 10.1155/2020/2953068

**Published:** 2020-03-18

**Authors:** Minghua Deng, Jingyuan Wang, Hao Wu, Menghao Wang, Ding Cao, Jinzheng Li, Yakun Wu, Jianping Gong

**Affiliations:** ^1^Department of Hepatobiliary Surgery, Second Affiliated Hospital of Chongqing Medical University, Chongqing 400010, China; ^2^Department of Hepatobiliary Surgery, Suining Central Hospital, Suining, Sichuan 629000, China

## Abstract

**Background:**

Liver ischaemia-reperfusion injury (IRI) remains a problem in liver transplantation. Interleukin-4 (IL-4) has been found to reduce liver IRI, but the exact mechanism remains unclear.

**Methods:**

Donor livers were infused with recombinant IL-4 or normal saline during cold storage, and the hepatocellular apoptosis and the inflammatory response were detected. The effect of IL-4 treatment on Kupffer cells (KCs) polarization and expression of the STAT6-JMJD3 pathway was evaluated *in vivo* and *in vitro*. KCs in donor livers were depleted by clodronate liposome treatment or JMJD3 was inhibited by GSK-J4 before liver transplantation to determine whether the protective effect of IL-4 treatment was dependent on KCs.

**Results:**

IL-4 treatment decreased sALT and sAST levels and alleviated hepatocellular apoptosis and inflammation at 6 h after liver transplantation. IL-4 treatment induced KCs alternatively activated (M2) polarization *in vitro*. KCs in donor livers were depleted by clodronate liposome treatment or JMJD3 was inhibited by GSK-J4 before liver transplantation to determine whether the protective effect of IL-4 treatment was dependent on KCs. *in vivo* and *in vitro*. KCs in donor livers were depleted by clodronate liposome treatment or JMJD3 was inhibited by GSK-J4 before liver transplantation to determine whether the protective effect of IL-4 treatment was dependent on KCs.

**Conclusions:**

IL-4 treatment-induced KCs M2 polarization was dependent on the STAT6-JMJD3 pathway and protected liver grafts from IRI after liver transplantation.

## 1. Introduction

With better disease control in patients before liver transplantation (LT), refined operative techniques, better organ preservation, and immunotherapy, the survival rate of recipients has improved by 60% in the last 20 years. LT has become one of the most important treatments for patients suffering from end-stage liver diseases, such as hepatic failure, liver cirrhosis, and hepatic malignancies [1]. However, liver ischaemia-reperfusion injury (IRI) remains inevitable and is one of the major reasons for increased liver injury during LT, which contributes to approximately 10% of early graft failure after transplantation [2]. Liver IRI is a dynamic process with complicated mechanisms, such as sterile inflammation, oxidative stress, hepatocyte apoptosis, and activation of Kupffer cells, which begins during hepatic ischaemia and is further strengthened during reperfusion [3]. However, despite obvious clinical improvement, the mechanisms that are responsible for liver IRI are not well understood, and further studies and new strategies to alleviate liver IRI are needed.

Kupffer cells (KCs), which account for 80–90% of all macrophages in mammalian organisms, play a key role in maintaining liver homeostasis in normal and pathological conditions, including liver IRI [4]. During the early stage of liver IRI, KCs are stimulated by damage-associated molecular patterns (DAMPs), high mobility group box 1 (HMGB1), and lipopolysaccharide (LPS) and then initiate excessive inflammation by releasing inflammatory cytokines and reactive oxygen species (ROS), which result in a positive feedback loop that increases the inflammatory cascade and leads to serious liver damage [5, 6]. Depending on the cell phenotype, macrophages can be divided into proinflammatory classically activated (M1) and anti-inflammatory alternatively activated (M2) macrophages [4]. M1 macrophages are characterized by the upregulation of proinflammatory cytokines, chemokines, and prostaglandins, such as IL-6, TNF-*α*, and IL-1*β*, to eliminate pathogens and facilitate the T helper type 1 (Th1) response. M2 macrophages are involved in the T helper type 2 (Th2) response and secrete IL-10 and TGF-*β* to reduce inflammation and promote tissue repair and they have been observed in spinal cord damage [7], wound skin healing [8], and acute renal injury [9]. Recently, multiple studies have shown that polarizing KCs to M2 phenotype contributes to increased liver transplantation tolerance and reduced IRI [5, 10]. Therefore, inducing M2 KCs may be essential to the treatment of liver IRI after liver transplantation.

Interleukin-4 (IL-4), mainly produced by Th2 cells, B cells, basophils, eosinophils, and NKT cells, is the prototypical direct inducer of M2 macrophages [11]. IL-4 phosphorylates STAT6, which translocates into the nucleus and regulates the expression of specific M2 macrophage genes. Jumonji domain containing 3 (JMJD3), also known as KDM6b, one of the Jumonji C (JmjC) domain protein family members, catalyses the demethylation of trimethylated lysine 27 on histone H3 (H3K27me3) [12], which is located on the promoter and/or enhancer of some genes and suppresses their transcriptional activity [13]. After stimulation with IL-4, STAT6 induces the expression of JMJD3 by directly binding to its promoter, and JMJD3 then decreases the H3K27me3 of M2 marker genes such as Chi3l3, Rentnla, Ym1, and Arg-1 [14, 15]. Therefore, we hypothesised that, in a rat liver transplantation model, IL-4 treatment could induce KCs M2 polarization through STAT6-JMJD3 pathway and alleviate inflammatory response and IRI after liver transplantation.

## 2. Materials and Methods

### 2.1. Animals and Liver Transplantation Models

Sprague Dawley rats (SD) (male, 250–300 g) were purchased from Chongqing Medical University Experimental Animal Centre (Chongqing, China). All rats were housed in an SPF level room at 24°C and 60% humidity with a 12 h light/dark cycle, and food and water were freely provided. Experiments were performed with the approval of the Animal Care and Use Committee of the Second Affiliated Hospital of Chongqing Medical University. Orthotopic liver transplantation was performed according to modified Kamada's two-cuff technique [16]. All surgical procedures followed the aseptic principle. The liver grafts were preserved in 4°C UW solution for 18 h prior to the further liver transplantation. The success rate of establishing a liver transplantation model was 100%. Details of the surgery can be found in our previous publication [17].

### 2.2. Donor Treatment

The rats were randomly divided into the Sham group (*n* = 6), Liver Transplantation group (LT, *n* = 6), Liver Transplantation + Normal Saline treatment group (LT + NS, *n* = 6), Liver Transplantation + IL-4 treatment group (LT + IL-4, *n* = 6), Liver Transplantation + Clodronate liposome treatment group (LT + CL, *n* = 6), and Liver Transplantation + GSK-J4 treatment group (LT + GSK-J4, *n* = 6). In the Sham group, the rats received an abdominal incision and exposure of the liver vascular. In the LT group, the rats received liver transplantation with no additional treatment. In the LT + NS group, the livers of donor rats were continuously infused with NS via portal vein (PV) cannulation using a syringe pump for 1 h at the beginning of cold storage. In the LT + IL-4 group, the livers of donor rats were continuously infused with recombinant rat IL-4 (Protech, USA) in a dose of 5 ng/kg/min via portal vein (PV) cannulation using a syringe pump for 1 h at the beginning of cold storage. In the LT + CL group, the donor rats were injected intraperitoneally with 50 mg/kg CL (78-BI, Shanghai, China) 24 h before liver transplantation. In the LT + GSK-J4 group, the donor rats were injected intraperitoneally with 5 mg/kg GSK-J4 (MCE, USA) for 3 consecutive days before liver transplantation. For tissue and serum examination, the rats were sacrificed 6 h after liver transplantation, which was the peak of hepatocellular damage in this model, and serums and liver tissues were collected and stored at −80°C.

### 2.3. Liver Function and Histological Examination

Serum alanine transaminase (sALT) and aspartate aminotransferase (sAST) were measured by an autoanalyser (Beckman CX7, USA). Liver tissues were fixed with 10% formalin, embedded in paraffin, sectioned at 5 *µ*m, placed on glass slides, stained with haematoxylin and eosin, and observed by microscopy. The severity of IRI was graded using the Suzuki criteria [18].

### 2.4. KCs Isolation and Culture

KCs were isolated from the liver by collagenase type IV (Sigma, USA) digestion and discontinuous density gradient centrifugation [19]. The purified KCs were cultured in RPMI 1640 containing 10% FBS and 1% streptomycin and penicillin.

### 2.5. Western Blot Analysis

KCs or liver tissue homogenates were lysed in RIPA lysis buffer (Beyotime, China). The concentration of total protein was measured by a BCA kit (Beyotime, China). A total of 30 *µ*g of protein sample was loaded and electrophoresed in 12.5% or 7.5% sodium dodecyl sulfate polyacrylamide gels and transferred to polyvinylidene fluoride membranes. The membranes were blocked with 5% skim milk at room temperature for 1 h and then incubated with JMJD3 (Introverge, USA), H3K27me3, STAT6, p-STAT6, P65, p-P65, BCL-2, BaX, cleaved caspase3, and GAPDH (CST, USA) primary antibodies at 4°C overnight. Next, the membranes were incubated with horseradish peroxidase-conjugated secondary antibody at room temperature for 1 h and stained with enhanced chemiluminescence reagents.

### 2.6. Cell Immunofluorescence

The cultured KCs from liver allografts were fixed in 4% paraformaldehyde for 20 min, treated with 0.4% Triton X-100 for 5 min, and then blocked with 3% BSA at 4°C overnight. The samples were incubated with JMJD3 antibody at 4°C overnight and then incubated with fluorescein isothiocyanate (DyLight488)–labelled goat anti-rabbit IgG (1 : 200) in darkness for 1 h. Images were taken by fluorescence electron microscopy (Olympus DX51, Japan).

### 2.7. Flow Cytometry (FCM) and Flow Imaging

KCs were collected and suspended as single cells (1 × 10^6^/ml) with PBS. Anti-F4/80-FITC and anti-CD206-PE (Bioscience, USA) were used to stain the KCs in darkness for 2 h, and then the cells were washed 3 times with PBS and resuspended in 500 *µ*l PBS. Flow cytometric data were acquired using Amnis Flowsight (Millipore, USA) and analysed by FlowJo 7.6.5 software.

### 2.8. Quantitative Real-Time Polymerase Chain Reaction

Total RNA was extracted from KCs lysed in TRIzol (Takara, Dalian, China) and then reverse-transcribed into cDNA by using the PrimeScript™ RT reagent Kit with gDNA Era-ser (Takara, Japan). The PCR assay was performed on a Bio-Rad CFX ConnectTM Real-Time System (Bio-Rad, Hercules, CA, USA). The following primers were used: Retnla (forward: 5′-GTCCCCAATGACAGCCCCTTTC-3′, reverse: 5′-CCACCCAGCACCACACTGATTG-3′); iNOS (forward: 5′-AGCAGGCACACGCAATGATGG-3′, reverse: 5′-GGACACCATGAGCACGGAAAGC-3′); TNF-*α* (forward: 5′-CGCCACGAGCAGGAATGAGAAG-3′, reverse: 5′-GGAAGCGTACCTACAGACTATC-3′); IL-1*β* (forward: 5′-AAATGAACCGAGAAGTGGTGTT-3′, reverse: 5′-TTCCATATTCCTCTTGGGGTAGA-3′); IL-6 (forward: 5′-GTTCTCTGGGAAATCGTGGA-3′, reverse: 5′-TGTACTCCAGGTAGCTA-3′); and GAPDH (forward: 5′ -TCAACGGGGGACATAAAAGT-3′, reverse: 5′-TGCATTGTTTTACCAGTGTCAA-3′). The relative expression was calculated using the ΔΔCq method.

### 2.9. TdT-Mediated dUTP Nick End Labelling (TUNEL) Assay

Apoptotic cells were detected by using the Apoptosis Detection Kit III, FITC (Keygen, China), following the instruction. Briefly, liver sections were treated with proteinase K for 30 minutes at 37°C and then treated by biotin-11-dUTP and TdT enzyme for 60 minutes at 37°C after being washed by PBS. These sections were further incubated by Streptavidin-Fluorescein for 30 minutes at 37°C. Images were obtained under a fluorescence microscope (Olympus DX51, Japan).

### 2.10. siRNA Transfection in KCs

Lipo8000™ transfection reagent (Beyotime, China) was used to transfect JMJD3 siRNA to KCs according to the instruction. The concentration of siRNA was 20 *µ*m. Transfected KCs were incubated with new culture medium for further treatment. Western blot was conducted to determine the silencing efficiency.

### 2.11. Hoechst33342 and Propidium Iodide (PI) Staining

Apoptotic Cell Hoechst33342/PI Detection Kit (Keygen, China) was used to detect the late apoptotic and necrotic cells according to the instruction. Briefly, hepatocytes were stained with Hoechst33342 (10 *μ*g/mL) for 15 minutes at 37°C, then washed by PBS, and stained by PI (5 *μ*g/mL) for 15 minutes at 37°C. The Hoechst and PI were excited at 352 and 488 nm, respectively. Hoechst33342 positive cells (blue) and PI-positive cells (red) were observed under a fluorescence electron microscopy (Olympus DX51, Japan).

### 2.12. Cell Viability Assay

Cell viability was measured using the Cell Counting Kit-8 (CCK8) (MCE, USA) according to the instruction. Briefly, KCs in 96-well plates were incubated with 100 *μ*L medium containing 10 *μ*L CCK8 reaction liquid at 37°C for 2 h. The absorbance at 450 nm was determined by a microplate spectrophotometer.

### 2.13. Statistical Analysis

All the data were presented as the mean ± SD. GraphPad Prism 7 software was used for all statistical analyses. The differences of gene expression, protein expression, liver function test, counting of TUNEL-positive cells, and counting of PI-positive cells across multiple parameters were analysed by one-way analysis of variance (one-way ANOVA test) followed by a Bonferroni post hoc test. Value of *p* < 0.05 was considered statistically significant differences.

## 3. Results

### 3.1. IL-4 Treatment on Donor Livers Alleviated IRI after Liver Transplantation

**Figure 1:**
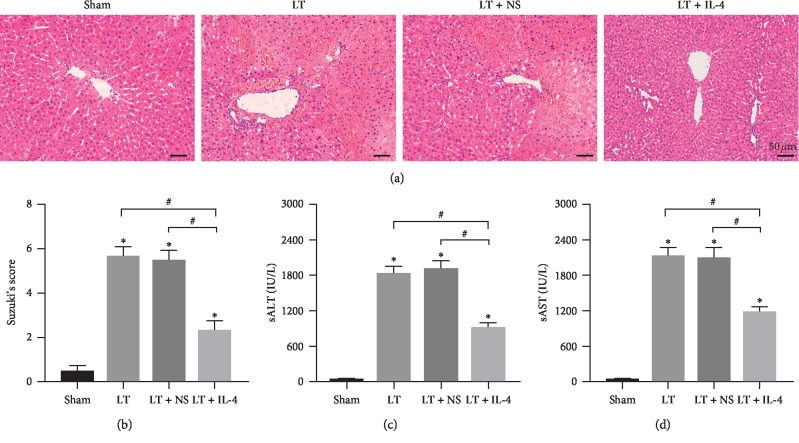
IL-4 treatment on donor livers alleviated IRI after liver transplantation. (a, b) Representative images of haematoxylin and eosin staining of liver grafts at 6 h after liver transplantation (original magnification, ×200) and Suzuki's histological grading of liver IRI (*n* = 6/group). (c, d) Levels of sAST and sALT were measured at 6 h after liver transplantation (*n* = 6/group). Data are shown as mean ± SD, ^*∗*^*p* < 0.05*vs.* the Sham group, ^#^*p* < 0.05*vs.* the LT + IL-4 group.

To explore whether IL-4 treatment could attenuate rat liver IRI after liver transplantation (LT), liver and serum samples were collected at 6 hours after liver transplantation, the peak of hepatocellular damage in this model [20]. Compared with the Sham group, Liver Transplantation caused obvious liver injury (Figure 1(a)). In the IL-4 + LT group, IL-4 treatment showed attenuated areas of sinusoidal congestion, hepatocellular necrosis, vacuolization, and neutrophil infiltration as compared with the LT and LT + NS groups (Figure 1(a)). These results were consistent with Suzuki's histological grading of hepatocellular damage (Figure 1(b)) and the depressed sALT and sAST levels (Figures 1(c) and 1(d)). Therefore, recombinant rat IL-4 treatment on the donor livers during cold storage alleviated liver IRI at 6 h after liver transplantation.

### 3.2. IL-4 Treatment on Donor Livers Suppressed Apoptosis and Inflammation Induced by IRI

**Figure 2:**
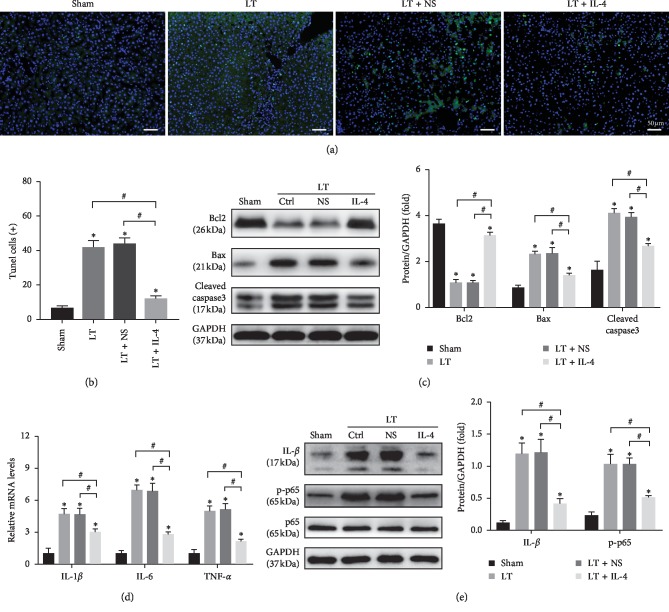
IL-4 treatment on donor livers suppressed apoptosis and inflammation induced by IRI. (a, b) Representative images of TUNEL-stained hepatocellular apoptosis in liver grafts at 6 h after liver transplantation (original magnification, ×200) and quantification of TUNEL-positive cells (*n* = 6/group). (c) The protein levels of Bcl2, Bax, and cleaved caspase3 at 6 h after liver transplantation were tested by Western blot. (d) The mRNA levels of proinflammatory factors (IL-1*β*, IL-6, and TNF-*α*) in the liver graft at 6 h after liver transplantation were detected by qRT-PCR. (e) The protein expression level of IL-1*β*, p-p65, and p65. GAPDH served as an internal control and was used for normalization. Data are shown as mean ± SD, ^*∗*^*p* < 0.05*vs.* the Sham group, ^#^*p* < 0.05*vs.* the LT + IL-4 group.

As apoptosis and sterile inflammation play a key role in liver IRI after liver transplantation, we measured the hepatocellular apoptosis and inflammatory response at 6 h after liver transplantation in the Sham, LT, LT + NS, and LT + IL-4 groups. TUNEL staining showed that liver transplantation caused obvious hepatocellular apoptosis compared with the Sham group and that IL-4 treatment significantly suppressed hepatocellular apoptosis compared with the LT and LT + NS group (Figures 2(a) and 2(b)). Moreover, the results of Western blot showed that the expression of proapoptotic proteins Bax and cleaved caspase3 was decreased and the expression of antiapoptotic protein Bcl2 was dramatically increased in the LT + IL-4 group (Figure 2(c)). To evaluate the inflammatory response, qRT-PCR was applied to detect the mRNA levels of IL-1*β*, IL-6, and TNF-*α* in liver tissues. The results showed that IL-4 treatment decreased the levels of these inflammatory factors compared with the LT and LT + NS groups at 6 h after liver transplantation (Figure 2(d)). Consistent with these results, IL-4 treatment also decreased the protein levels of IL-1*β* and p-p65 (Figure 2(e)). These results confirmed that IL-4 treatment on liver donors during cold storage attenuated liver apoptosis and inflammatory responses at 6 h after liver transplantation.

### 3.3. IL-4 Treatment Induced KCs M2 Polarization and Increased the Expression of JMJD3 *In Vivo*

**Figure 3:**
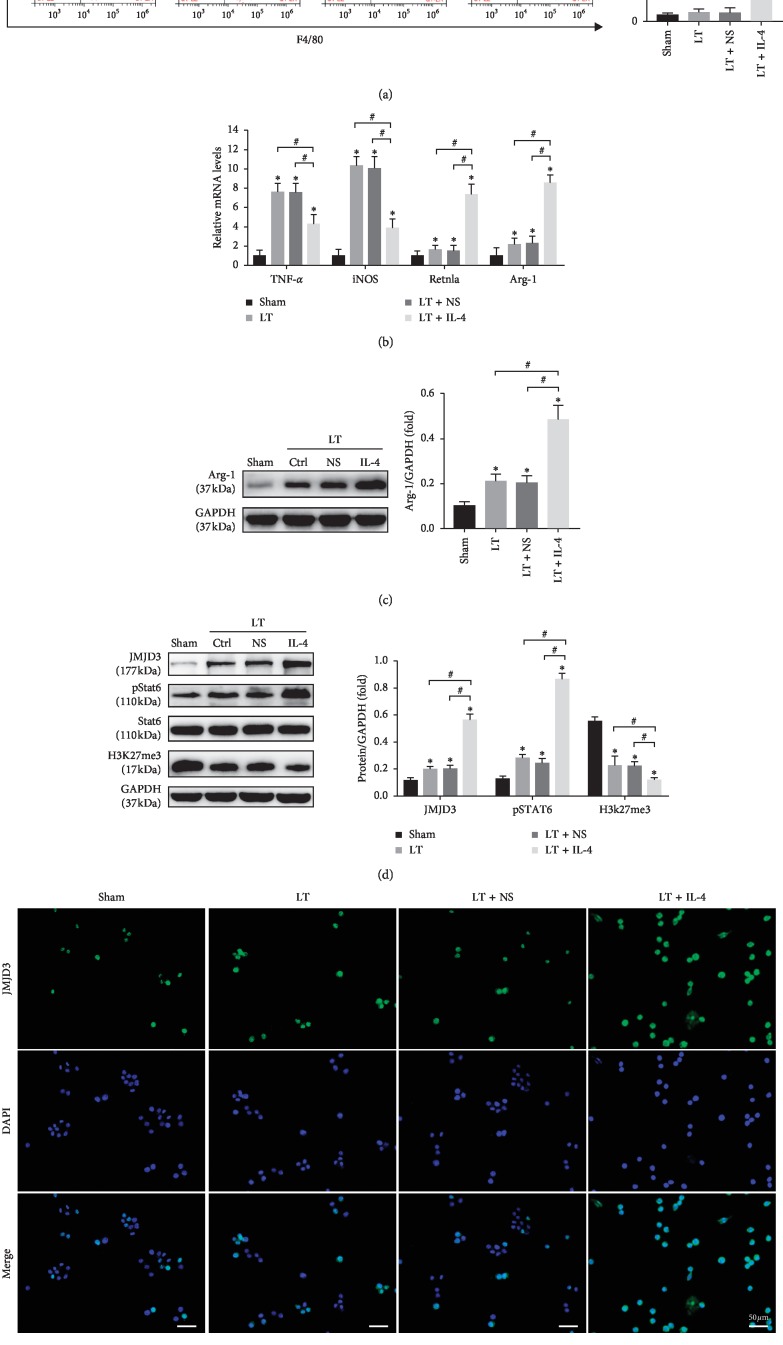
IL-4 treatment induced KCs M2 polarization and increased the expression of JMJD3 *in vivo*. KCs were isolated from the Sham group, LT group, LT + NS group, and LT + IL-4 group at 6 h after liver transplantation. (a) The ratio of F4/80^+^CD206^+^ KCs was measured by flow cytometry. (b) The mRNA levels of KC marker genes, including TNF-α, iNOS, Arg-1, and Retnla, were detected by qRT-PCR. (c) The protein expression level of Arg-1 was tested by Western blot (*n* = 3/group). (d) The protein expression levels of JMJD3, p-STAT6, STAT6, and H3K27me3 in KCs were measured by Western blot. GAPDH served as an internal control and was used for normalization (*n* = 3/group). (e) The expression of JMJD3 (green) in KCs was detected by cell immunofluorescence, and DAPI (blue) was used for nuclear staining. Data are shown as mean ± SD, ^*∗*^*p* < 0.05*vs.* the Sham group, ^#^*p* < 0.05*vs.* the LT + IL-4 group.

KCs play a key role in the regulation of homeostasis and immunity at the early stage of liver IRI. To determine whether the treatment of donor liver with IL-4 had an impact on KCs polarization, KCs were isolated from allografts at 6 h after liver transplantation. Compared with the LT and LT + NS groups, the ratio of M2 KCs was dramatically increased in the LT + IL-4 group as indicated by the result of FCM (Figure 3(a)). The mRNA levels of M2-specific marker genes, including Arg-1 and Retnla, were upregulated, and M1-specific marker genes, including TNF-*α* and iNOS, were downregulated (Figure 3(b)). Moreover, the protein level of Arg-1 also significantly increased in the LT + IL-4 group (Figure 3(c)). These results showed that IL-4 treatment on liver donors induced KCs M2 polarization. The STAT6-JMJD3 pathway plays a key role in M2 macrophage polarization [14]. To investigate KCs M2 polarization through the STAT6-JMJD3 pathway, STAT6, JMJD3, and H3K27me3 in KCs were detected. We found that the phosphorylation status of STAT6 (p-STAT6) and JMJD3 were increased in the LT, LT + NS, and LT + IL-4 groups, but were dramatically increased in the LT + IL-4 group (Figure 3(d)). H3K27me3, which is specifically catalysed by JMJD3 to H3k27me2/1, was decreased (Figure 3(d)). Cell immunofluorescence also demonstrated the same results (Figure 3(e)). According to these results, we thought that IL-4 treatment on liver donors activated the STAT6-JMJD3 pathway in KCs and induced KCs M2 polarization.

### 3.4. IL-4 Treatment-Induced KCs M2 Polarization Was Dependent on STAT6-JMJD3 Pathway *In Vitro*

**Figure 4:**
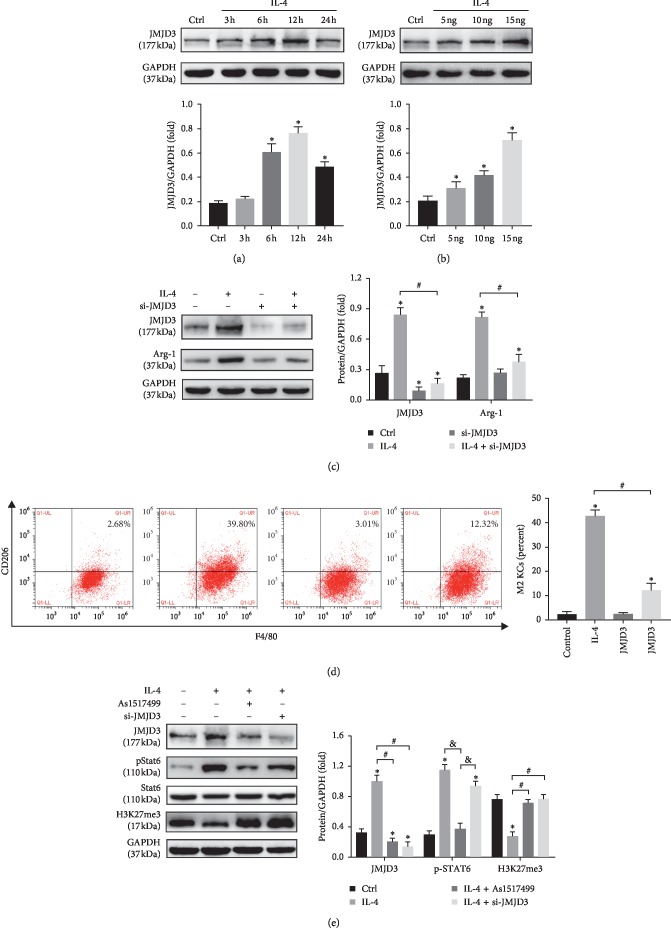
IL-4 induced KCs M2 polarization was dependent on the STAT6-JMJD3 pathway *in vitro*. KCs were isolated from untreated livers, and IL-4-treated KCs were pretreated with siRNA-JMJD3 or the STAT6 inhibitor As1517499. (a, b) KCs were incubated for the indicated times or with different concentrations of IL-4 for 12 h; then the protein expression level of JMJD3 was detected by Western blot (*n* = 3/group). (c, d) The ratio of F4/80^+^CD206^+^ KCs was measured by flow cytometry, and the expression of Arg-1 and JMJD3 was analysed by Western blot (*n* = 3/group). (e) The protein expression levels of JMJD3, p-STAT6, and H3K27me3 were detected by Western blot. GAPDH served as an internal control and was used for normalization (*n* = 3/group). Data are shown as mean ± SD, ^*∗*^*p* < 0.05*vs.* the control group, ^#^*p* < 0.05*vs.* the IL-4 group, ^&^*p* < 0.05*vs.* the IL-4 + As1517499 group.

To further confirm whether the KCs M2 polarization induced by IL-4 was dependent on the STAT6-JMJD3 pathway, KCs isolated from rats were treated with IL-4. We found that IL-4 could significantly induce the expression of JMJD3 at a dose of 15 ng/ml for 12h as indicated by the results of Western blot (Figures 4(a) and 4(b)). JMJD3 in KCs was knocked down by using RNA interference (siRNA-JMJD3) (Figure 4(c)). IL-4 treatment led to an elevated expression of Arg-1 and the ratio of M2 KCs (Figures 4(c) and 4(d)) which were dramatically blocked by JMJD3 knockdown. As1517499, an effective STAT6 inhibitor, abolished the elevated expression of p-STAT6 and JMJD3 induced by IL-4. However, JMJD3 knockdown had little impact on p-STAT6 induced by IL-4 treatment (Figure 4(e)). The changes in H3K27me3 were opposite to that of JMJD3 (Figure 4(e)). Taking together, these observations suggested that IL-4-induced KCs M2 polarization was dependent on the STAT6-JMJD3 pathway and the catalytic activity of JMJD3.

### 3.5. JMJD3 Knockdown Weakened the Anti-inflammatory and Antiapoptotic Effects of IL-4 *In Vitro*

**Figure 5:**
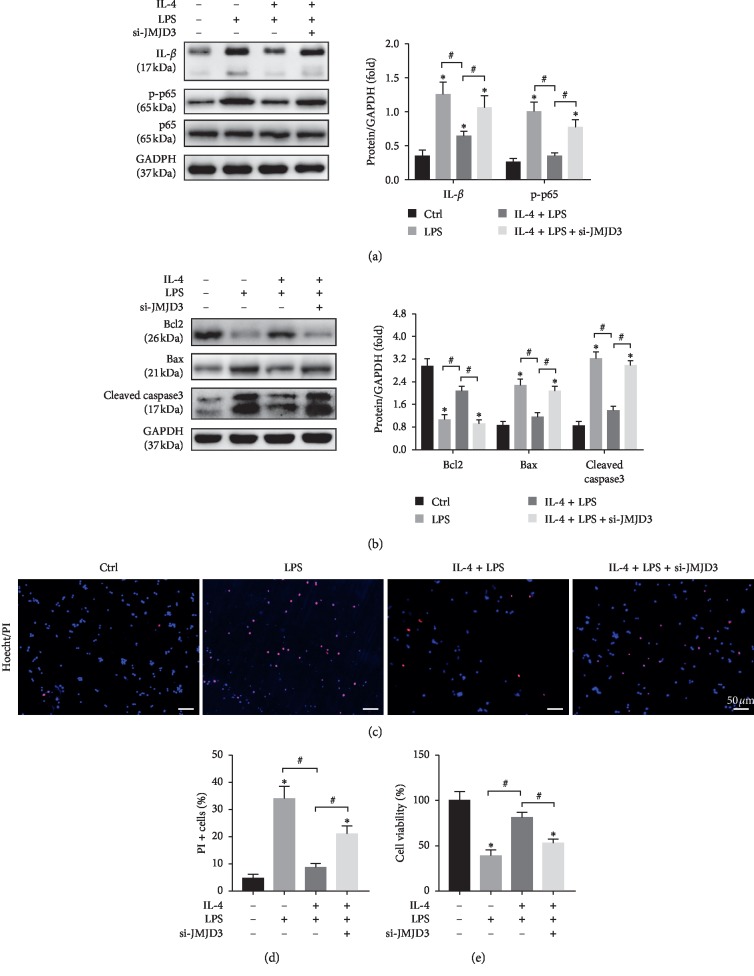
JMJD3 knockdown weakened the anti-inflammatory and antiapoptotic effects of IL-4 *in vitro*. KCs and primary hepatocytes were isolated from untreated livers. KCs were treated with 100 ng/mL LPS for 24 h with or without treatment of 15 ng/mL IL-4 for 12 h The supernatant of the KCs was collected and used to stimulate primary hepatocytes. (a) The protein expression levels of IL-*β*, p-p65, p65, and GAPDH were detected by Western blot. (b) The protein expression levels of Bcl2, Bax, cleaved caspase3, and GAPDH were detected by Western blot (*n* = 3/group). GAPDH served as an internal control and was used for normalization (*n* = 3/group). (c) Representative images of Hoechst33342 staining for late apoptosis and necrotic cells: hoechst333424 (blue) and PI (red). (d) Quantification of PI-positive stained cells (*n* = 3/group). (e) Cell viability was detected by CCK8 (*n* = 3/group). The data are presented as the mean ± SD, ^*∗*^*p* < 0.05*vs.* the control group, ^#^*p* < 0.05*vs.* the IL-4 + LPS group.

To investigate the impact of JMJD3 knockdown on IL-4 treatment, LPS was used to induce sterile inflammation in KCs. The protein expression of IL-1*β* and p-p65 increased in LPS-treated KCs, which was attenuated by IL-4 pretreatment (Figure 5(a)). However, the anti-inflammatory effect of IL-4 was partly blocked by JMJD3 knockdown (Figure 5(a)), because IL-4 treatment on donor livers alleviated hepatocellular apoptosis (Figures 2(a)–2(c)). To determine whether IL-4-induced M2 KCs participated in this process, the supernatants of KCs were collected and used to stimulate primary hepatocytes. The result of Western blot demonstrated that IL-4 pretreatment reduced LPS-induced hepatocyte apoptosis, which was abolished by JMJD3 knockdown (Figure 5(b)). Hoechst33342-PI staining showed that the number of late apoptotic and necrotic cells (PI-positive) was decreased by IL-4 treatment, which was abolished by JMJD3 knockdown (Figures 5(c) and 5(d)). JMJD3 knockdown also blocked the IL-4-induced improvement of cell viability, as shown by the CCK8 assays (Figure 5(e)).

### 3.6. Liver IRI Inhibition Induced by IL-4 Is due to KCs M2 Polarization

**Figure 6:**
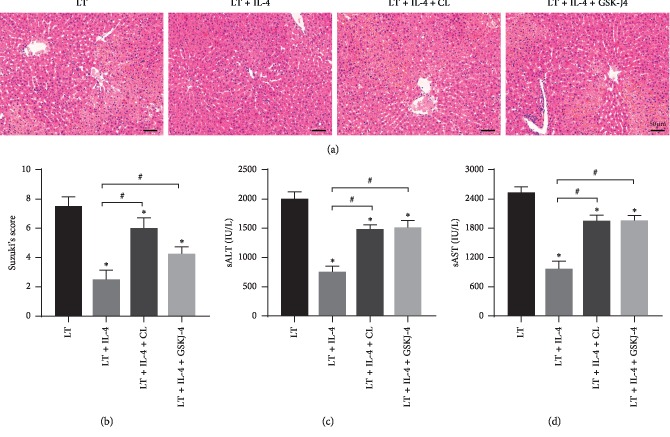
Liver IRI inhibition induced by IL-4 is due to KCs M2 polarization. KCs in the donor livers were depleted by clodronate liposome treatment, or donor liver JMJD3 was inhibited by GSK-J4 before liver transplantation. (a, b) Representative images of haematoxylin and eosin staining of liver graft at 6 h after liver transplantation (original magnification, ×200) and Suzuki's histological grading of liver IRI (*n* = 6/group). (c, d) Levels of sAST and sALT were measured at 6 h after liver transplantation (*n* = 6/group). The data are shown as mean ± SD, ^*∗*^*p* < 0.05*vs.* the Sham group, ^#^*p* < 0.05*vs.* the LT + IL-4 group.

IL-4 treatment on donor livers alleviated liver IRI and led to KCs M2 polarization. To further determine that the protection against liver IRI induced by liver transplantation was due to KCs M2 polarization, KCs in the LT + IL-4 + CL group were depleted by clodronate liposome (CL), and JMJD3 in the LT + IL-4 + GSK-J4 group was inhibited by the pretreatment of GSK-J4, an effective JMJD3 inhibitor which had been widely used [21]. HE staining showed that depletion of KCs abolished the protective effect of IL-4 treatment, and inhibition of JMJD3 also obviously blocked the protective effect of IL-4 treatment (Figure 6(a)). The Suzuki scores and sALT and sAST levels gave the same results (Figures 6(b)–6(d)).

## 4. Discussion

Liver ischaemia-reperfusion injury (IRI) is an important factor affecting the prognosis of liver transplantation, and inhibiting liver IRI is an effective method to reduce acute rejection and improve the survival rate after liver transplantation [22]. In the present study, we found that infusion of recombinant IL-4 in donor livers during cold storage alleviated liver IRI. IL-4 treatment improved liver function as well as decreased inflammation and hepatocellular apoptosis. Mechanistically, infusion of recombinant IL-4 in the donor livers promoted activation of STAT6-JMJD3 pathway in KCs and polarized KCs to the M2 phenotype. Inhibition of JMJD3 blocked the protective effect of IL-4 treatment *in vitro* and *in vivo*.

The liver harbours the largest proportion of macrophages among all solid organs, and the macrophages derived from foetal liver and embryonic yolk are named KCs [23]. KCs have high phagocytic and lysosomal activity and can remove potentially harmful endogenous and extracellular compounds, such as dying red cells, microorganisms, and metabolic waste. Perhaps because of their continuous exposure to gut-derived antigens and bacterial endotoxins, KCs exhibit a tolerogenic phenotype in the healthy livers [24]. Similar to macrophages, KCs have been classified as proinflammatory M1 KCs and anti-inflammatory M2 KCs according to the differences in phenotype and secretion of inflammatory cytokines [25]. In acute infection, overdose of hepatotoxic drugs, and liver IRI, KCs are activated by DAMPs via TLR3, TLR4, and TLR9 and polarized to the M1 phenotype, which induces proinflammatory cascades and infiltration of monocytes, neutrophils, and T lymphocytes, which exacerbate liver injury [26–28]. In our previous studies, we found that blocking the M1 KC polarization or promoting the KCs M2 polarization reduced liver IRI and organ rejection [18, 29, 30].

IL-4 is a cytokine that is mainly produced by Th2 cells, mediates type 2 inflammation, and induces M2 macrophage polarization [31], which is characterized by anti-inflammation [32]. Some studies have shown that IL-4 deficiency exacerbates acute kidney injury and cerebral ischaemia injury, with an increase in M1 macrophages and a decrease in M2 macrophages [9, 33]. IL-4 is essential for tissue protection from acute organ injury. Kato et al. [34] found that administration of IL-4 before and after liver warm ischaemia-reperfusion suppressed TNF-*α*, neutrophil cell accumulation, and liver injury. Furthermore, in a rat liver transplantation model, Wang et al. [35] suggested that IL-4 treatment on the donor rat by intraperitoneal injections increased the immunosuppressive cells, including M2 macrophages and IDO-expressing NK cells, and reduced IRI and acute rejection, while IL-4 treatment on the recipients after liver transplantation led to exacerbation of allograft injury and rejection.

Whether KCs were involved in this process and whether the protection of IL-4 treatment on donor livers was dependent on KCs have not been investigated yet. In the present study, to investigate how KCs respond to IL-4, recombinant IL-4 was infused into donor livers during cold storage; therefore, IL-4 only affected cells in the donor livers without promoting direct recruitment of bone marrow-derived cells. Depletion of KCs in the donor livers by clodronate liposome treatment decreased the protective effect of IL-4, as indicated by the results of sALT, sAST, and HE staining. Therefore, the protection of IL-4 treatment against liver IRI after liver transplantation was mainly dependent on KCs in the donor livers.

Trimethylation of histone H3 lysine 27 (H3K27me3), one of posttranslational modifications, is closely associated with gene silencing. JMJD3, a member of the JMJC family, has been reported to demethylate H3K27me3 and activate gene expression. The role of JMJD3 in the regulation of inflammation is complicated. JMJD3 can be activated by NF-*қ*B pathway induced by LPS and promote the expression of inflammatory factors [36]. However, some studies have found that STAT6-JMJD3 pathway plays an important role in the M2 macrophage polarization induced by IL-4 [14] and chitin [37], which can suppress inflammatory response *in vitro*. What role the STAT6-JMJD3 pathway plays when the tissues or organs are under injures has not been reported. To the best of our knowledge, our study is the first time to document the beneficial impact of the STAT6-JMJD3 pathway in liver transplantation. We found that IL-4 treatment activated the STAT6-JMJD3 pathway *in vivo* and *in vitro*. And JMJD3 knockdown decreased KCs M2 polarization induced by IL-4 and reduced the anti-inflammatory and antiapoptotic effects, which suggested that the protective effect of IL-4 was dependent on JMJD3.

As TLR-4-NF-*κ*B pathway in KCs is a classical signalling pathway and is activated during liver IRI, which is the main reason for sterile inflammation and hepatocellular apoptosis [38], we also detected the impact of IL-4 on these *in vivo* and *in vitro*. IL-4 treatment suppressed the inflammatory response and cell apoptosis induced by IRI and LPS. We found that the expression of JMJD3 was mainly induced by IL-4, but not LPS. Treatment with IL-4 alleviated LPS-induced inflammation, and this phenomenon was similar to those of another report [39]. We hypothesised that IL-4 pretreatment-induced KCs M2 polarization depended on STAT6-JMJD3 pathway, which then attenuated inflammatory response caused by the following liver injury or LPS treatment *in vivo* and *in vitro*. Ultimately, apoptosis induced by those factors was suppressed. But, how STAT6 or NF-*κ*B determines the gene binding site of JMJD3 and whether JMJD3 can regulate the polarization of KCs in a demethylase-independent manner require further study.

## 5. Conclusion

The present study showed that IL-4 treatment on donor livers induced KCs M2 polarization and protected liver grafts from IRI by regulating the inflammatory response and hepatocellular apoptosis. The mechanism is related to the activation of STAT6-JMJD3 pathway and suppression of TLR-4-NF-*κ*B pathway. The detailed process of how JMJD3 regulates the polarization of KCs requires further study. This study showed that IL-4 treatment on donor livers may be an effective strategy and that STAT6-JMJD3 may be a new therapeutic target for preventing liver IRI after liver transplantation.

## Data Availability

All the data used to support the findings of this study are included within the article.
